# Activation of nuclear factor kappa B pathway and reduction of hypothalamic oxytocin following hypothalamic lesions

**DOI:** 10.15761/JSIN.1000114

**Published:** 2016-01-29

**Authors:** Christian L. Roth, Gabrielle D’Ambrosio, Clinton Elfers

**Affiliations:** 1 Division of Endocrinology, Department of Pediatrics, University of Washington, USA; 2 Center for Integrative Brain Research, Seattle Children’s Research Institute, USA

**Keywords:** hypothalamic lesion, obesity, hyperphagia, inflammation, nuclear factor kappa B, oxytocin

## Abstract

**Background:**

Hypothalamic obesity (HO) occurs in patients with tumors and lesions in the medial hypothalamic region. In this study, a hyperphagic rat model of combined medial hypothalamic lesions (CMHL) was used to test which specific inflammatory molecules are involved.

**Methods:**

In order to target specific homeostatic medial hypothalamic nuclei (arcuate, ventromedial, and dorsomedial nuclei), male Sprague-Dawley rats (age of 8 weeks, ~250 g body weight) received four electrolytic lesions or sham surgery. Post-surgery food intake and weight changes were tracked and hypothalamic gene expression for inflammatory molecules as well as anorexigenic peptide oxytocin 7 days and 7 months post-surgery were tested.

**Results:**

Seven days post-surgery, average food intake increased by 23%, and body weight gain had increased by 68%. Toll-like 4 receptor/nuclear factor–κB (TLR4/NF–κB)—pathway was specifically activated in the mediobasal hypothalamus (MBH), resulting in 3-fold higher tumor necrosis factor (TNF)-α, 10-fold higher interleukin (IL) 1-β mRNA levels, and higher expression of suppression of cytokine signaling (SOCS) 3, while oxytocin mRNA levels were significantly reduced in CMHL rats versus sham surgery rats 7 days post-surgery. At 7 months, inflammation was less stimulated in MBH of CMHL rats compared to 7 days post-surgery and SOCS 3 as well as oxytocin mRNA levels were comparable between the two groups.

**Conclusion:**

Medial hypothalamic lesions are associated with strong post-surgery hyperphagia and activation of TLR4/NF–κB—pathway as well as reduced expression of oxytocin in the hypothalamus.

## Introduction

Childhood obesity of any cause is a major risk factor for adult CVD and premature death [1]. Excessive weight gain frequently occurs in patients with hypothalamic tumors and lesions, a disorder designated as hypothalamic obesity (HO). HO syndrome is characterized by fatigue, decreased physical activity, hyperphagia, decreased satiety, and severe obesity. All features are frequently seen in patients suffering from HO due to craniopharyngioma (CP) [2,3] as well as other causes of hypothalamic dysfunction and damage: other suprasellar tumors, inflammation, and genetic syndromes [4–7]. Most efforts to treat HO have disappointing long-term success rates [2,3].

Patients with CP often develop severe obesity with metabolic syndrome and have a much higher overall mortality rate and cardiovascular mortality rate than the general population [8–10]. Recognized risk factors for severe obesity include large hypothalamic tumors or lesions affecting several medial and posterior hypothalamic nuclei that impact satiety signaling pathways [11–14]. Structural damage in these nuclei often lead to hyperphagia, rapid weight gain, central insulin and leptin resistance, decreased sympathetic activity, low energy expenditure, and increased energy storage in adipose tissue [2,15]. Recently, our group developed a novel rat model of combined medial hypothalamic lesions (CMHL) to study the pathogenesis of HO and test potential drugs for obesity treatment and prevention [2,16]. This rodent model reproduces clinical features of hypothalamic obesity in patients with craniopharyngioma. We have shown in humans and rodents that large hypothalamic tumors or lesions affecting several medial hypothalamic regions including the arcuate (ARC), ventromedial (VMN), and dorsomedial (DMN) nuclei, lead to a more severe phenotype of HO and melanocortin (MC) deficiency compared to smaller lesions and lesions of single nuclei [2,16–22]. The risk for gaining excess weight is particularly high during the immediate period following hypothalamic surgery [23–25]. In our previous studies we also demonstrated hyperinsulinemia and high leptin serum levels indicating leptin resistance as observed in patients with CP [16,26]. The goal of the current study was to investigate the pathophysiology of hyperphagia observed in HO or CP and specifically to test in our CMHL model if hyperphagia is related to hypothalamic inflammation and which specific inflammatory pathways are involved.

## Materials and methods

### Animals

All procedures performed were approved by the Institutional Animal Care and Use Committee at the Seattle Children’s Research Institute and were in accordance with the NIH Guide for the Care and Use of Laboratory Animals. Young adult male Sprague Dawley rats, weighing 250 to 265 g, were purchased from Charles River Laboratory (Wilmington, MA, USA). Animals were individually housed on a 12hr/12hr light/dark cycle (lights on at 07:00 h) in a temperature (23°C) and humidity (50% ± 10%) controlled room. Ad libitum access to regular chow (5053 Pico Lab Rodent Diet, Purina LabDiet, Richmond, CA, USA) and water was provided. Following surgery, the animals’ body weight (BW) and food intake (FI) were recorded twice daily, at begin of light and dark cycle respectively. Lee adiposity index [BW-1/3/snout to anus length (mm)] measures were taken pre surgery and at termination as an indicator of total adiposity.

### Hypothalamic lesion

**Figure 1:**
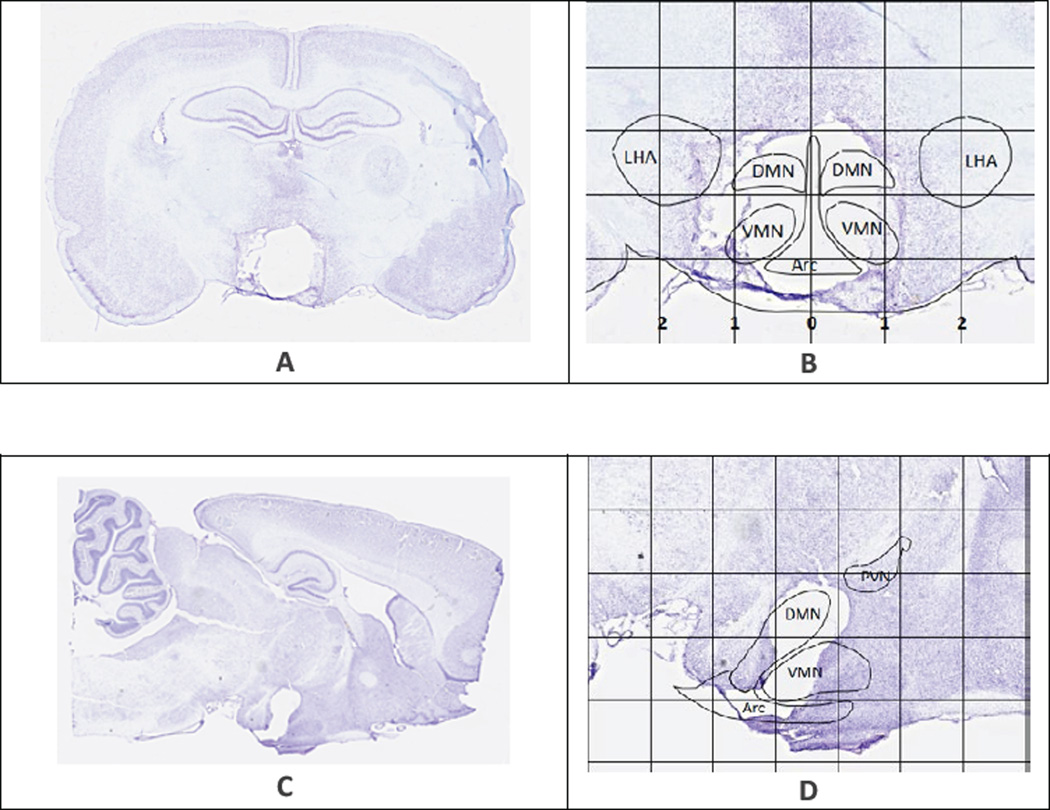
Representative brain sections showing the extent of lesion in two CMHL rats. Rat brains were harvested 15 and 13 days after lesion respectively. Grid lines in image **B and D** are measured in millimeters. **A, B**: Lateral lesion coordinates measure 0.5 mm to the left and right of sagittal midline (0 mm), descending directly into the DMN and VMN. Radius of the electrode also encompasses the ARC, creating a cohesive zone of ablation at the lesion target area. **C, D**: Dorsal and ventral lesion targets (−8.7 mm and −10 mm depth) are distinguishable in sagittal section. PVN is outlined in image **D**. The PVN is located at about Bregma −1.80 to Bregma −2.12 mm, which is rostral from the lesion target slice at Bregma −2.80 mm, leaving the PVN intact. Projection of CMHL brain nuclei based on the atlas of Paxinos and Watson [27].

As published before [16,21,22], animals were placed under a surgical-plane of anesthesia (isoflurane/oxygen mix, 3.5% induction, 2–2.5% throughout the procedure) and were mounted in a Kopf stereotaxic frame (Tujunga, CA). The upper incisor bar was initially set 3.3 mm below the interaural line and adjusted as necessary to align bregma and lambda in the same horizontal plane. Lesions were targeted to the ARC, VMN, and DMN through the placement of an insulated stainless steel electrode at stereotaxic coordinates based on our previous study [16]. Specifically, an anodal electric current (110V, 1.7 mA for 15 seconds; no current for sham group; 5–7 animals per treatment group) was passed through the tip of the electrode while placed 2.6 mm posterior to the bregma, 0.5 mm lateral to the midsagittal line, and both 10 mm (ARC) and 8.6 mm (VMN/DMN) ventral from the bregma-lambda plane according to the atlas from Paxinos and Watson [27]. Please see examples in Figure 1 for details.

### Histological examination

One extra set of 7 animals was used for brain section studies during the first 2 weeks after the lesion. These animals were anesthetized and perfused transcardially with 4% paraformaldehyde and could therefore not be used for gene expression studies in the brain. Brains were harvested and cut in 25 µm thick tissue slices using a cryostat followed by staining with cresyl violet. These slides were then imaged at 20× resolution using the Hamamasu Nano Zoomer (Hamamasu Photonics, Hamamasu City, Japan).

### Tissue sampling and gene expression studies

Using freshly frozen brains, the mediobasal hypothalamus (MBH) was cut as a rectangular tissue block extending from Bregma coordinates −1.92 to −4.4 mm, 1.4 mm lateral from midline, and 2 mm depth measured from base ventrally according to the atlas from Paxinos and Watson [27].

Total RNA was extracted and purified using RNeasy kit (Qiagen, Venlo, Netherlands) from brain tissues. RNA quality was assessed using a NanoDrop 1000 (Thermo Fisher, Waltham, MA), and cDNA was synthesized using iScript cDNA synthesis kit (Bio-Rad, Hercules, CA). Real-time qRT-PCR reactions were performed on an ABI Prism 7300 (Applied Biosystems, Foster City, CA) using iTaq SYBR Green Supermix with ROX (Bio-Rad, Hercules, CA). Nontemplate controls were incorporated into each PCR run. Specific mRNA levels of all genes of interest were normalized to housekeeping gene glyceraldehyde-3-phosphate dehydrogenase (GAPDH) and expressed as changes normalized to controls (sham lesions). See Table 1 for all primers of quantitative RT-PCR.

The mRNA levels for inflammatory molecules (toll-like receptor (TLR)-4; nuclear factor kappa B (NF–κB); interleukin (IL)-1β; tumor necrosis factor (TNF)-α; and suppression of cytokine signaling (SOCS)3) were quantified in the MBH. IL-1β was also quantified in the temporal cortex as a control. The mRNA levels of key anorexigenic peptide oxytocin were quantified in the MBH as well.

### Statistical analysis

Statistical analyses were performed using GraphPad Prism Software (La Jolla, CA USA). Outcome variables between study groups were compared using Student’s t-tests for continuous variables and single intervention comparisons (for instance sham surgery vs. lesion). In all instances, p<0.05 was considered significant.

## Results

**Figure 2:**
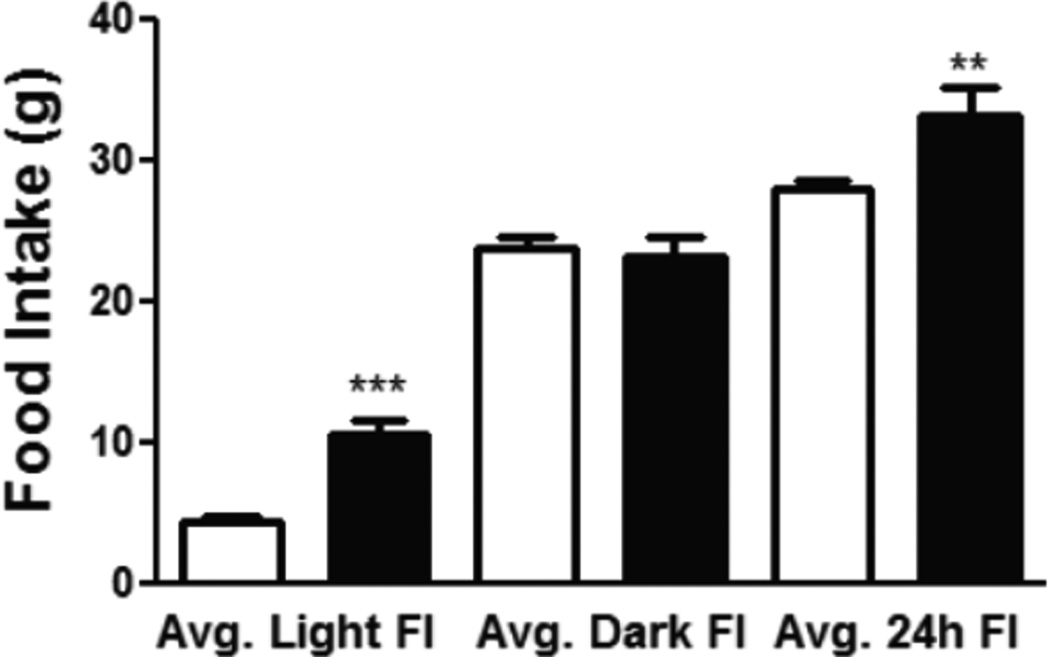
Food intake in rats following CMHL. Averaged food intake during first 7 days following surgery is shown during light and dark cycle as well as 24 h in CMHL (black bars) vs. sham surgery (white bars) rats. **p< 0.01, ***p< 0.001.

**Figure 3:**
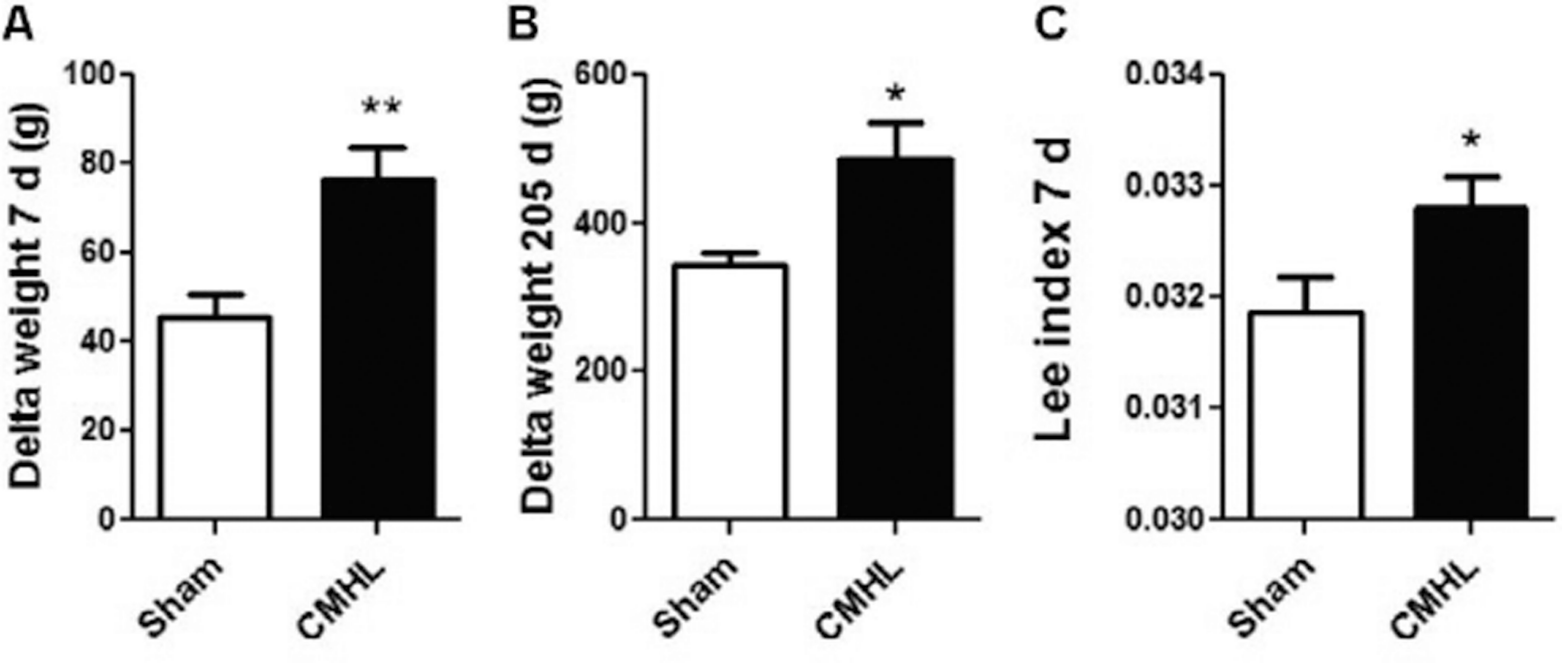
Changes in body weight and adiposity following surgery. Body weight changes 7 days **(A)** and 7 months (205 d) (**B**) after surgery. Adiposity index shows increased adiposity already 7 d after surgery (**C**). CMHL (black bars) vs. sham surgery (white bars) rats are shown. *p< 0.05, **p< 0.01.

Histological examination shows that the CMHL model results in a large medial hypothalamic lesion including the ARC, VMN, and DMN as shown in coronal and sagittal sections of the brains (Figure 1). In one extra set of 7 animals, which was used for brain section studies, the extent of the hypothalamic damage following CMHL was comparable in different rats. In CMHL rats, food intake was significantly increased in CMHL versus sham surgery rats during the light cycle resulting in an increased 24 h food intake during the first 7 d following surgery (Figure 2). However at 7 months, food intake was not significantly different compared to sham surgery rats (CMHL 31.3 ± 2.4 *vs.* sham 28.7 ± 0.6 g/d, p=0.342). CMHL lesion resulted in significantly increased weight gain and adiposity assessed by increased Lee index already 7 d post-surgery (Figures 3a and 3c, respectively). The stronger weight gain continued in CMHL rats showing a marked difference to sham surgery rats 7 months post-surgery (Figure 3b).

**Figure 4:**
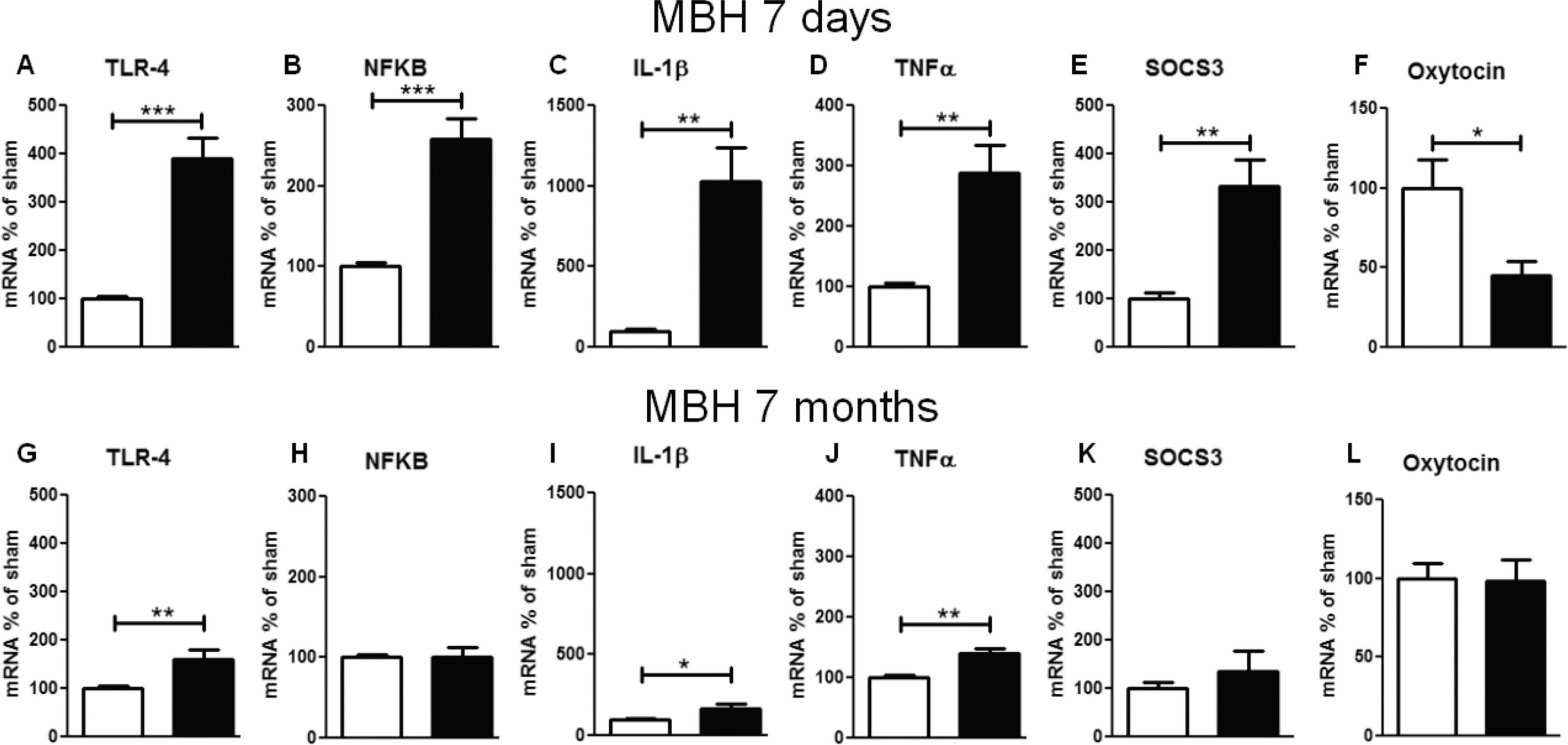
Gene expression of inflammatory parameters in MBH of CMHL (black bars) vs. sham-surgery controls (white bars), 7 d (**A–F**) and 7 months(**G–L**) post-surgery; mRNA levels of TLR-4 (**A, G**), NFkB (**B, H**), IL-1β (**C, I**), TNF-α (**D, J**), SOCS3 (**E, K**), and oxytocin (**F, L**) related to GAPDH are shown. **/***p< 0.01/0.001.

Regarding the gene expression of inflammatory molecules 7 d after CMHL, mRNA levels for TLR4, TNFα, NF–κB IL-1β, and SOCS3 were increased in MBH (including the ARC, VMN, and DMN), while mRNA levels of oxytocin were significantly reduced in the MBH of CMHL *vs.* sham-surgery rats. However, 7 months after surgery, stimulation of inflammatory pathways was less compared to 7 days post-surgery and there were no differences between the groups for oxytocin mRNA levels (Figure 4). IL-1β mRNA levels were not different in cerebral cortex, 7 d post-surgery, CMHL vs. sham surgery (data not shown).

## Discussion

The data from this study confirm our previously published results demonstrating that large medial hypothalamic lesions result in excessive short and long-term weight gain as well as transient hyperphagia with increased food intake particularly during the light cycle indicating a disrupted circadian rhythm [16,20]. The excess weight gain and hyperphagia occurs directly after surgery, already resulting in a significantly increased adiposity index 7 d post-surgery. At this time the strongly activated inflammatory NF-κB pathway is most likely related to surgery, whereas the slightly increased expression of few inflammatory molecules at 7 months is likely related to the severe obesity the animals achieved at this late time point.

Brain inflammatory responses are a hallmark of CP [26,28–30]. Previous studies demonstrated massively increased interleukin expression in CP tissue as well as increased IL-1α and TNF-α in CP cyst fluid [31]. What remains unknown, however, is the role of inflammation in tumor- or surgery-related excess weight gain and food intake. Our gene expression data demonstrate activation of the TLR4/NF–κB—pathway in CMHL rat, resulting in activation of TNF-α and IL1-β specifically in the MBH. Activation of similar pathways has also been associated with hypothalamic inflammation due to over-nutrition resulting in central insulin and leptin resistance [32–37]. In addition, inflammation induced upregulation of SOCS-3, a marker of leptin and insulin resistance [38], can result in impaired ability of satiety signals, such as cholecystokinin, to activate neurons in the hindbrain and reduce food intake [39]. Therefore, these changes not only affect hypothalamic signaling, but also the regulation of energy homeostasis by downstream neurons [32–34,40], and may include reward pathways [41,42].

Insights into the specific inflammatory pathways are important for identifying potential targets for novel pharmacotherapies directed towards specific biochemical pathways. Based on these results, one could postulate that interacting with these pathways may yield promising options to reduce food intake, especially considering the specific agents already available. For instance, food intake could be suppressed by central suppression of NF-kB pathway as shown in diet-induced obese rodent models [43–45]. Another option could be an IL receptor antagonist that crosses the blood brain barrier [46]. Future studies will show if postsurgical hyperphagia is dependent upon hypothalamic stimulation of the NF–κB pathway and if hyperphagia and leptin resistance can be attenuated by specific anti-inflammatory drugs targeted to this pathway.

Disruption of feeding circuits by damage to medial hypothalamic nuclei by tumor, surgery or irradiation, has the potential to increase hunger by unopposed activation of orexigens from the lateral hypothalamus, or by blocking response to adiposity signals such as leptin and proopiomelanocortin in the arcuate nucleus from the medial hypothalamus. In either case, the result can involve unopposed activation of the LHA, and thereby orexigenic peptides (MCH, hypocretin/orexin), or inhibition of anorexigenic peptides in the PVN (CRF, TRH, oxytocin), finally resulting in increased food intake and decreased energy expenditure. Orexin A and B derive from the same precursor, prepro-orexin (identical with prepro-hypocretin), which is selectively expressed in the LHA; both peptides stimulate food intake [47]. Oxytocin is produced primarily in the PVN and supraoptic nucleus [48], but is also produced in several other brain areas involved in energy homeostasis such as the LHA [49,50]. Leptin activates PVN oxytocin neurons through a melanocortin-4 receptor dependent mechanism whereas leptin resistance is associated with downstream impairments in oxytocin release [51–53]. Thus, CPs, by virtue of their hypothalamic location, have the potential to disrupt energy homeostasis at many levels, resulting in a complex clinical picture of HO syndrome characterized by severe obesity associated with leptin-resistance, fatigue, hyperphagia, impaired satiety, decreased sympathetic tone, and low energy expenditure [15,54–58].

Identification of effective strategies to treat HO is still one of the major challenges in the post-surgical care of CP patients. Here we have demonstrated activation of the TLR4/NF-κB pathway and induction of SOCS3 in the MBH while oxytocin expression was reduced after hypothalamic lesions which could give important insights for future studies on the field. However we are aware that there are some limitations of the study that should kept in mind when interpreting the data. First, the gene expression studies have been performed only at two different time points that are well apart so we don’t know how long the strong activation of inflammatory pathways will last beyond the 7 day time point. Second, to test the relationship between activation of TLR4/NF-κB pathway and satiety neuropeptides, it would be necessary to test the effects of different anti-inflammatory agents on the expression of a variety of satiety neuropeptides in brain areas associated with energy homeostasis. This was beyond the scope of this project, but could be tested in the future.

In summary, using a rat brain lesion model that recapitulates unique clinical features seen in obese CP patients, we identified robust activation of the TLR4/NF-κB pathway at an early time point (7 d) but not late time point (7 months) after surgery. Induction of SOCS3 and reduction of oxytocin expression might explain leptin and insulin resistance as well as hyperphagia that are observed in CMHL rats already 7 d post-surgery [16,38] and that are also key clinical features in CP patients [25,59].

## Figures and Tables

**Table 1:** Primers Used for Real-Time PCR Studies (5’ – 3’), Gen Bank numbers (Rattus norvegicus), and Amplicon Lengths.

	Accession Number	Forward	Reverse	bp
GAPDH	NR_003722.1	TGCACCACCAACTGCTTAGC	GGCATGGACTGTGGTCATGAG	87
IL-1β	NM_031512.2	CACCTCTCAAGCAGAGCACAG	GGGTTCCATGGTGAAGTCAAC	79
NF-κB	NM_001008349.1	TGGGCAGACACGGGTGGTGA	ATTTGGGGCAGAGCGTGGGC	165
Oxytocin	NM_012996.3	CGGTGGATCTCGGACTGAAC	TAGCAGGCGGAGGTCAGAG	91
SOCS3	NM_053565.1	CCTCCAGCATCTTTGTCGGAAGAC	TACTGGTCCAGGAACTCCCGAATG	99
TCR-4	NM_019178.1	GGGGCAACCGCTGGGAGAGA	AACCAGCGGAGGCCGTGAGA	95
TNFα	NM_012675.3	GAAAAGCAAGCAACCAGCCA	CGGATCATGCTTTCCGTGCTC	106

## Abbreviations

ARC: arcuate nucleus
BW: body weight
CMHL: combined medial hypothalamic lesions
CRF: Corticotrophin-releasing hormone
CVD: cardiovascular disease
DMN: dorsomedial nucleus
FI: food intake
GABDH: glyceraldehyde-3-phosphate dehydrogenase
HO: hypothalamic obesity
IL: interleukin
MBH: mediobasal hypothalamus
MCH: Melanin-concentrating hormone
NF-kB: nuclear factor-kB
SOCS3: suppression of cytokine signaling 3
TRH: Thyrotropin-releasing hormone
TLR-4: toll-like 4 receptor
TNF-α: tumor necrosis factor-α
VMN: ventromedial nucleus

## References

[1] Steinberger J, Daniels SR (2003). Obesity, insulin resistance, diabetes, and cardiovascular risk in children: an American Heart Association scientific statement from the Atherosclerosis, Hypertension, and Obesity in the Young Committee (Council on Cardiovascular Disease in the Young) and the Diabetes Committee (Council on Nutrition, Physical Activity, and Metabolism). Circulation.

[2] Roth CL (2011). Hypothalamic obesity in patients with craniopharyngioma: profound changes of several weight regulatory circuits. Front Endocrinol (Lausanne).

[3] Müller HL (2014). Craniopharyngioma. Endocr Rev.

[4] Goldstone AP (2006). The hypothalamus, hormones, and hunger: alterations in human obesity and illness. Prog Brain Res.

[5] Zegers D, Van Hul W, Van Gaal LF, Beckers S (2012). Monogenic and complex forms of obesity: insights from genetics reveal the leptin-melanocortin signaling pathway as a common player. Crit Rev Eukaryot Gene Expr.

[6] Cai D (2013). Neuroinflammation and neurodegeneration in overnutrition-induced diseases. Trends Endocrinol Metab.

[7] Farooqi IS (2006). Monogenic human obesity syndromes. Prog Brain Res.

[8] Mong S, Pomeroy SL, Cecchin F, Juraszek A, Alexander ME (2008). Cardiac risk after craniopharyngioma therapy. Pediatr Neurol.

[9] Srinivasan S, Ogle GD, Garnett SP, Briody JN, Lee JW (2004). Features of the metabolic syndrome after childhood craniopharyngioma. J Clin Endocrinol Metab.

[10] Simoneau-Roy J, O’Gorman C, Pencharz P, Adeli K, Daneman D (2010). Insulin sensitivity and secretion in children and adolescents with hypothalamic obesity following treatment for craniopharyngioma. Clin Endocrinol (Oxf).

[11] Roth CL, Eslamy H, Werny D, Elfers C (2015). Semiquantitative analysis of hypothalamic damage on MRI predicts risk for hypothalamic obesity. Obesity (Silver Spring).

[12] Bereket A, Kiess W, Lustig RH, Muller HL, Goldstone AP (2012). Hypothalamic obesity in children. Obes Rev.

[13] Elowe-Gruau E, Beltrand J, Brauner R, Pinto G, Samara-Boustani D (2013). Childhood craniopharyngioma: hypothalamus-sparing surgery decreases the risk of obesity. J Clin Endocrinol Metab.

[14] Müller HL, Gebhardt U, Teske C, Faldum A, Zwiener I (2011). Post-operative hypothalamic lesions and obesity in childhood craniopharyngioma: results of the multinational prospective trial KRANIOPHARYNGEOM 2000 after 3-year follow-up. Eur J Endocrinol.

[15] Lustig RH (2011). Hypothalamic obesity after craniopharyngioma: mechanisms, diagnosis, and treatment. Front Endocrinol (Lausanne).

[16] Roth CL, Blevins JE, Ralston M, Elfers C, Ogimoto K (2011). A novel rodent model that mimics the metabolic sequelae of obese craniopharyngioma patients. Pediatr Res.

[17] Müller HL, Bueb K, Bartels U, Roth C, Harz K (2001). Obesity after childhood craniopharyngioma--German multicenter study on pre-operative risk factors and quality of life. Klin Padiatr.

[18] Roth CL, Gebhardt U, Müller HL (2011). Appetite-regulating hormone changes in patients with craniopharyngioma. Obesity (Silver Spring).

[19] Roth CL, Enriori PJ, Gebhardt U, Hinney A, Müller HL (2010). Changes of peripheral alpha-melanocyte-stimulating hormone in childhood obesity. Metabolism.

[20] Elfers C, Ralston M, Roth CL (2011). Studies of different female rat models of hypothalamic obesity. J Pediatr Endocrinol Metab.

[21] Elfers CT, Roth CL (2011). Effects of methylphenidate on weight gain and food intake in hypothalamic obesity. Front Endocrinol (Lausanne).

[22] Elfers CT, Simmons JH, Roth CL (2012). Glucagon-like peptide-1 agonist exendin-4 leads to reduction of weight and caloric intake in a rat model of hypothalamic obesity. Horm Res Paediatr.

[23] Müller HL, Heinrich M, Bueb K, Etavard-Gorris N, Gebhardt U (2003). Perioperative dexamethasone treatment in childhood craniopharyngioma--influence on short-term and long-term weight gain. Exp Clin Endocrinol Diabetes.

[24] Müller HL, Emser A, Faldum A, Bruhnken G, Etavard-Gorris N (2004). Longitudinal study on growth and body mass index before and after diagnosis of childhood craniopharyngioma. J Clin Endocrinol Metab.

[25] Roth CL, Eslamy H, Werny D, Elfers C (2015). Semiquantitative analysis of hypothalamic damage on MRI predicts risk for hypothalamic obesity. Obesity (Silver Spring).

[26] Roth CL (2015). Hypothalamic Obesity in Craniopharyngioma Patients: Disturbed Energy Homeostasis Related to Extent of Hypothalamic Damage and Its Implication for Obesity Intervention. J Clin Med.

[27] Palombi O, Shin JW, Watson C, Paxinos G (2006). Neuroanatomical affiliation visualization-interface system. Neuroinformatics.

[28] Mutlu LK, Woiciechowsky C, Bechmann I (2004). Inflammatory response after neurosurgery. Best Pract Res Clin Anaesthesiol.

[29] Martelli C, Iavarone F, Vincenzoni F, Rossetti DV, D’Angelo L (2014). Proteomic characterization of pediatric craniopharyngioma intracystic fluid by LC-MS top-down/bottom-up integrated approaches. Electrophoresis.

[30] Pettorini BL, Inzitari R, Massimi L, Tamburrini G, Caldarelli M (2010). The role of inflammation in the genesis of the cystic component of craniopharyngiomas. Childs Nerv Syst.

[31] Mori M, Takeshima H, Kuratsu J (2004). Expression of interleukin-6 in human craniopharyngiomas: a possible inducer of tumor-associated inflammation. Int J Mol Med.

[32] Thaler JP, Yi CX, Schur EA, Guyenet SJ, Hwang BH (2012). Obesity is associated with hypothalamic injury in rodents and humans. J Clin Invest.

[33] Thaler JP, Schwartz MW (2010). Minireview: Inflammation and obesity pathogenesis: the hypothalamus heats up. Endocrinology.

[34] Wisse BE, Schwartz MW (2009). Does hypothalamic inflammation cause obesity?. Cell Metab.

[35] Yang Q, Kim YS, Lin Y, Lewis J, Neckers L (2006). Tumour necrosis factor receptor 1 mediates endoplasmic reticulum stress-induced activation of the MAP kinase JNK. EMBO Rep.

[36] Yang L, Hotamisligil GS (2008). Stressing the brain, fattening the body. Cell.

[37] Denis RG, Arruda AP, Romanatto T, Milanski M, Coope A (2010). TNF-α transiently induces endoplasmic reticulum stress and an incomplete unfolded protein response in the hypothalamus. Neuroscience.

[38] El-Haschimi K, Pierroz DD, Hileman SM, Bjørbaek C, Flier JS (2000). Two defects contribute to hypothalamic leptin resistance in mice with diet-induced obesity. J Clin Invest.

[39] Savastano DM, Covasa M (2005). Adaptation to a high-fat diet leads to hyperphagia and diminished sensitivity to cholecystokinin in rats. J Nutr.

[40] Velloso LA (2009). The brain is the conductor: diet-induced inflammation overlapping physiological control of body mass and metabolism. Arq Bras Endocrinol Metabol.

[41] Horvath TL, Sarman B, García-Cáceres C, Enriori PJ, Sotonyi P (2010). Synaptic input organization of the melanocortin system predicts diet-induced hypothalamic reactive gliosis and obesity. Proc Natl Acad Sci U S A.

[42] Cazettes F, Cohen JI, Yau PL, Talbot H, Convit A (2011). Obesity-mediated inflammation may damage the brain circuit that regulates food intake. Brain Res.

[43] Posey KA, Clegg DJ, Printz RL, Byun J, Morton GJ (2009). Hypothalamic proinflammatory lipid accumulation, inflammation, and insulin resistance in rats fed a high-fat diet. Am J Physiol Endocrinol Metab.

[44] Zhang Y, Scarpace PJ (2006). Circumventing central leptin resistance: lessons from central leptin and POMC gene delivery. Peptides.

[45] Thaler JP, Choi SJ, Sajan MP, Ogimoto K, Nguyen HT (2009). Atypical protein kinase C activity in the hypothalamus is required for lipopolysaccharide-mediated sickness responses. Endocrinology.

[46] Fox E, Jayaprakash N, Pham TH, Rowley A, McCully CL (2010). The serum and cerebrospinal fluid pharmacokinetics of anakinra after intravenous administration to non-human primates. J Neuroimmunol.

[47] Jöhren O, Neidert SJ, Kummer M, Dominiak P (2002). Sexually dimorphic expression of prepro-orexin mRNA in the rat hypothalamus. Peptides.

[48] Douglas AJ, Johnstone LE, Leng G (2007). Neuroendocrine mechanisms of change in food intake during pregnancy: a potential role for brain oxytocin. Physiol Behav.

[49] Shi H, Bartness TJ (2000). Catecholaminergic enzymes, vasopressin and oxytocin distribution in Siberian hamster brain. Brain Res Bull.

[50] Blevins JE, Ho JM (2013). Role of oxytocin signaling in the regulation of body weight. Rev Endocr Metab Disord.

[51] Matarazzo V, Schaller F, Nédélec E, Benani A, Pénicaud L (2012). Inactivation of Socs3 in the hypothalamus enhances the hindbrain response to endogenous satiety signals via oxytocin signaling. J Neurosci.

[52] Münzberg H, Flier JS, Bjørbaek C (2004). Region-specific leptin resistance within the hypothalamus of diet-induced obese mice. Endocrinology.

[53] Maejima Y, Sedbazar U, Suyama S, Kohno D, Onaka T (2009). Nesfatin-1-regulated oxytocinergic signaling in the paraventricular nucleus causes anorexia through a leptin-independent melanocortin pathway. Cell Metab.

[54] Hochberg I, Hochberg Z (2010). Expanding the definition of hypothalamic obesity. Obes Rev.

[55] Harz KJ, Müller HL, Waldeck E, Pudel V, Roth C (2003). Obesity in patients with craniopharyngioma: assessment of food intake and movement counts indicating physical activity. J Clin Endocrinol Metab.

[56] Holmer H, Pozarek G, Wirfalt E, Popovic V, Ekman B (2010). Reduced energy expenditure and impaired feeding-related signals but not high energy intake reinforces hypothalamic obesity in adults with childhood onset craniopharyngioma. J Clin Endocrinol Metab.

[57] Roth CL, Hunneman DH, Gebhardt U, Stoffel-Wagner B, Reinehr T (2007). Reduced sympathetic metabolites in urine of obese patients with craniopharyngioma. Pediatr Res.

[58] Cohen M, Syme C, McCrindle BW, Hamilton J (2013). Autonomic nervous system balance in children and adolescents with craniopharyngioma and hypothalamic obesity. Eur J Endocrinol.

[59] Muller HL (2008). Childhood craniopharyngioma. Recent advances in diagnosis, treatment and follow-up. Horm Res.

